# Regional odontodysplasia: a report of unusual case with in-depth analysis

**DOI:** 10.1016/j.jobcr.2025.12.012

**Published:** 2026-01-15

**Authors:** Charles Béhot, Ophélie Cuisinier, Éden Serraf, Lisa Friedlander, Brigitte Vi-Fane, Pascal Garrec, Ali Nassif

**Affiliations:** a Biomedical Research in Oral Health, INSERM UMR1333, Université Paris Cité, 1 Rue Maurice Arnoux, Montrouge, 92120, France; b Department of Orthodontics, Pitié-Salpêtrière Hospital, 83, bd de l'hôpital, Paris, 75013, France; c Competences Center for Oral Diseases, Pitié-Salpêtrière Hospital, 83, bd de l'hôpital, Paris, 75013, France; d Department of Orthodontics, Bretonneau Hospital, 2 rue Carpeaux, Paris, 75018, France

**Keywords:** Regional odontodysplasia, Dental mineral density, Enamel hypoplasia, Micro-computed tomography (micro-CT)

## Abstract

**Background/purpose:**

Regional odontodysplasia (RO) is a rare developmental anomaly affecting dental tissue formation, typically confined to a single quadrant. It manifests through hypoplastic enamel, irregular dentin, short roots with open apices, and a characteristic “ghost-like” radiographic appearance. Its etiology remains unclear, with vascular, genetic and inflammatory factors proposed.

**Materials and methods:**

In this study, we report the case of a 15-year-old female with mandibular RO affecting canine, premolars, and molars, focusing on the clinical, radiographic, and microscopic analysis of the affected teeth, including mineral density evaluation.

**Results:**

The 2nd premolar and first and second molars (teeth 35, 36, 37) exhibited severe morphological defects and were extracted due to extreme root shortening (40 %, 90 %, and >95 % reduction), while alveolar bone height remained preserved. The canine and 1st premolar were affected to a lesser extent and were conserved upon orthodontic treatment plan. Scanning electron microscopy revealed slight decreases in the number and diameter of the dentinal tubules in the affected teeth compared with those in the control teeth. Contrary to many reported cases of RO, mineral density analysis of enamel and dentin revealed no significant differences between affected and normal teeth. The observations reported in this case suggest that resorptive changes, possibly influenced by inflammatory factors, could contribute to the observed root anomalies. The preservation of alveolar bone height provides a favourable prognosis for prosthetic rehabilitation.

**Conclusion:**

These findings help elucidate the structural and functional implications of ROs. This study highlights the importance of early diagnosis and multidisciplinary management for optimal outcomes.

## 1 Introduction

Regional odontodysplasia (RO) is an uncommon localized dental developmental anomaly, with approximately 200 cases reported globally as of 2023. ^1^ It is characterized by hypoplasia and hypocalcification of dental hard tissues and involves both ectodermal and mesodermal components. ^2^^,^^3^ It was first identified by Hitchin in 1934; the term “odontodysplasia” was introduced by Zegarelli in 1963, and Pindborg later added the prefix “regional” to reflect its localized nature.^2^^,^^4^^,^^5^ RO is often referred to as “ghost teeth” owing to its distinctive radiological appearance.

Clinically, RO typically affects multiple adjacent teeth within a quadrant. Most RO cases involve the maxilla, while mandibular involvement, particularly in the posterior region is extremely rare. ^3^^,^^6^ It is usually unilateral, rarely crossing the midline, and can involve both primary and permanent dentitions. The affected teeth are often smaller with underdeveloped roots and abnormal crown morphology, including irregular shapes, pits, or grooves. Hypoplasia and hypocalcification of enamel and dentin have also been reported. Discoloration ranging from yellow to brown is also common, alongside delayed or failed eruption.^6^^,^^7^ Radiographically, RO is characterized by poorly defined enamel and dentine layers, enlarged pulp chambers, short roots with open apices, and a “ghost-like” appearance. ^3^

Histological sections of teeth affected by RO show abnormalities in all dental tissues. The enamel is hypoplastic and hypocalcified, with an irregular prism structure. Dentine features include intertubular areas, irregular dentinal tubules, and an enlarged pre-dentine layer. ^2^^,^^7^ The pulp often contains calcifications, and follicular tissue may exhibit dysplastic changes. These defects contribute to the structural fragility of affected teeth, increasing their susceptibility to caries and bacterial infiltration. ^6^

The etiology of RO is unclear and multifactorial, involving local/systemic factors such as trauma, vascular anomalies, neural crest defects, metabolic disturbances, and genetic mutations, such as in the PAX9 gene. ^3^^,^^6^ Some cases are linked to teratogenic drugs or viral activation during tooth development. ^1^

Managing RO is challenging due to their complexity and variability. Treatment is often multidisciplinary and involves orthodontists and paediatric dentists, oral surgeons, and prosthodontists and ranges from conserving affected teeth to aid jaw development, to extractions with prosthetic rehabilitation.^7^ The decision largely depends on the patient's age, the severity of the condition, and aesthetic and functional considerations. In cases of severe structural compromise, such as teeth weakened by enamel and dentin hypoplasia, early extraction may prevent complications such as abscess or pain, whereas preserving noninfected teeth can maintain alveolar bone integrity and support normal occlusion. ^6^ Early diagnosis and customized treatment planning are vital for managing RO effectively. Further research is needed to elucidate its pathogenesis and improve therapeutic outcomes. Increased awareness among clinicians is essential for timely identification and intervention, ultimately enhancing the quality of life for affected patients.

In this article, we report an unusual case of regional odontodysplasia following surgery in an orthodontic department. Additionally, our study combines multiple analytical techniques, including high-resolution micro-computed tomography (micro-CT), scanning electron microscopy (SEM), and quantitative mineral density analysis with control comparisons. This integrated approach provides a detailed assessment of both the morphological and microstructural characteristics of affected teeth, contributing novel insights into RO pathology.

## 2 Case report

A 15-year-old female patient presented at the oral rare diseases centre and orthodontic department of La Pitié Salpêtrière Hospital. Clinical and radiographic evaluations revealed that mandibular lower left sector was affected. Three mandibular teeth—premolar 35 and molars 36 and 37—were severely affected (lower left mandible), whereas canine (33) and the 1st premolar (34) were affected to a lesser extent, exhibiting most of the RO-like features of anomalies and malformed short roots. Differential diagnoses include dentinogenesis imperfecta, dentin dysplasia and amelogenesis imperfecta. These diagnoses were excluded upon clinical and radiographic examination.

The patient's medical history was unremarkable, and there was no familial history of regional odontodysplasia or similar dental anomalies.

Written informed consent for participation, clinical treatment, and publication of anonymized clinical details and images was obtained from the patient and her legal guardian. The study was conducted in accordance with institutional ethical standards and the principles of the Declaration of Helsinki.

**Fig. 1:**
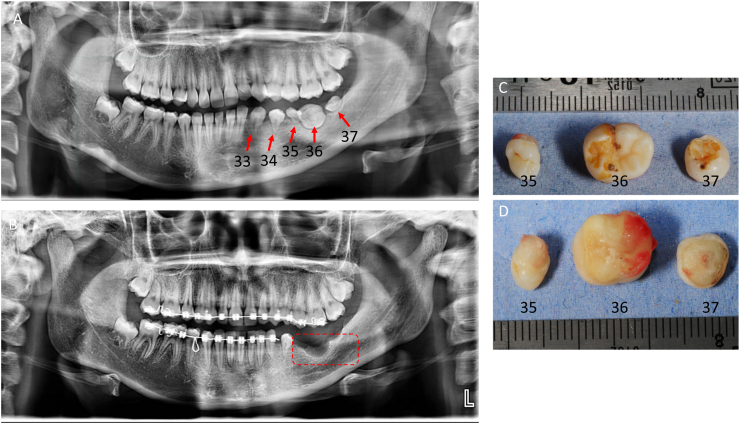
Panoramic radiograph showing abnormal development of teeth 33, 34, 35, 36, and 37, with notable defects in enamel and dentin (**A**). Following this radiographic assessment, the three most severely affected permanent teeth—one premolar (35) and 2 M (36 and 37)—were extracted for further analysis (**B**). Photographs of the extracted teeth show the occlusal aspect (**C**) and the roots aspect (**D**).

As part of her orthodontic treatment plan, the decision was made to preserve canine (33) and premolar (34) and to extract three other affected teeth to facilitate the orthodontic preparation required for subsequent prosthetic rehabilitation. This approach aims to optimize space management and alignment in the affected quadrant while preserving functional and aesthetic outcomes (Fig. 1).

The extracted teeth were subjected to microscopic analysis, with a focus on structural anomalies, and the mineral density of the dental tissues was evaluated to better understand the pathological alterations associated with RO.

## 3 Methods

### 3.1 Micro-CT acquisition and analysis

High-resolution micro-computed tomography (micro-CT) imaging was performed at the Life Imaging Facility (Plateforme des Imageries du Vivant, UMR-S 1333 Santé Orale, Université Paris Cité and Sorbonne Paris Nord, Inserm, Montrouge, France) using a Quantum FX X-ray micro-CT scanner (Rigaku Life Sciences, Revvity). Acquisition parameters included 90 kV voltage, 160 μA intensity, 10 mm field of view, 120-s scan time, and a voxel size of 20 μm^3^ (supplementary material 1).

Reconstructed images were processed using Analyze14® software (AnalyzeDirect, Mayo Clinic). Image noise was reduced via a 3D median filter (kernel size 3 × 3 × 3). Regions of interest (ROIs) were manually defined based on anatomical landmarks, specifically the enamel–dentine junction and the dentine core. Regions of interest (ROIs) were manually defined based on standardized anatomical landmarks, ensuring consistent assessment of comparable tooth structures in both affected and control samples. ROI selection was performed by an experienced operator and documented to enable replicability. Reference standards consisted of control teeth matched for age, dental type, and anatomical location. Control teeth were obtained from healthy premolars extractions performed in the context of orthodontic treatment (n = 3). These extractions involved teeth with no clinical or radiological signs of pathology and were selected to match the patient's age, dental type, and anatomical location as closely as possible. Image densities were scaled in Hounsfield Units (HU) during acquisition.

### 3.2 Scanning electron microscopy (SEM)

Extracted teeth were fixed in 2.5 % (v/v) glutaraldehyde (Sigma-Aldrich) in 0.1 M phosphate buffer (pH 7.2) at 4 °C for 24 h. Specimens underwent graded ethanol dehydration (30 %, 50 %, 70 %, 80 %, 90 %, 95 %, 100 %) with immersion for 5–15 min at each concentration. Teeth were sectioned using a diamond saw, preserving anatomical orientation. Surfaces were polished using silicon carbide abrasive papers of decreasing grit sizes (600, 800, 1000, 1200). Samples were etched with 37 % phosphoric acid (Merck) for 30 s, air dried for 24 h, and coated with a thin layer of gold-palladium using a sputter coater (model to be specified). SEM imaging was performed on a JSM-6400 (JEOL Ltd.) at 15 kV accelerating voltage and working distance between 28 and 55 mm.

### 3.3 Statistical analysis

Multiple unpaired t-tests were performed to compare all quantitative data of affected (RO) and control teeth, with the tooth as the statistical unit (averaging multiple regions of interest within each tooth). Correction for multiple comparisons was not applied due to the exploratory nature of this single-case study. P-values <0.05 are reported as indicative of significance.

## 4 Results

The extracted teeth exhibited most developmental anomalies characteristic of RO, including eruption disturbances, root malformations in both shape and number, and radicular dysplasia.

Crown anomalies were also noted, with the teeth appearing smaller than average. While the crowns of teeth 35 and 36 retained anatomical details typical of their counterparts, tooth 37 displayed significant deviations from the normal anatomy expected for this tooth. It presents premolar like characteristics with only two cuspids (vestibular and lingual). Additionally, agenesis of the germ of the third molar (tooth 38) was observed while it is present in the contralateral side (48).

Despite the severe lack of radicular formation, vertical alveolar bone growth has been relatively achieved. No other dental anomalies were identified in the maxillary arch or the right mandibular right hemi-arcade. However, a slight reduction in root length was noted for the upper left lateral incisor compared with its contralateral counterpart (Fig. 1).

### 4.1 Morphological analysis: crown, root, and pulp space

Microcomputed tomography (micro-CT) and 3D reconstructed imaging revealed significant morphological anomalies in the affected teeth. The crowns of teeth 35 and 36 retained partial anatomical similarity to their respective counterparts, whereas tooth 37 displayed severe dysplasia, deviating significantly from the expected morphology of a mandibular second molar.

**Fig. 2:**
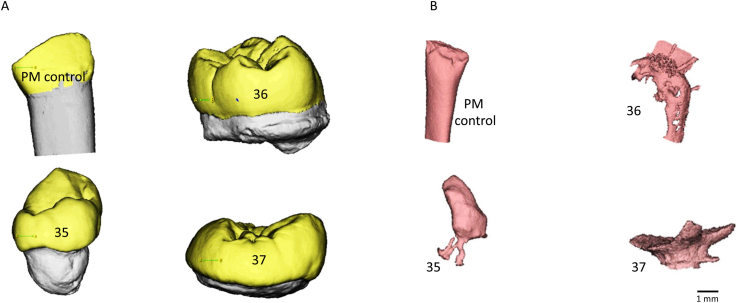
**SEM and 3D reconstruction of micro-CT images of the RO and control teeth.** SEM images of teeth affected by regional odontodysplasia (B, C, and D) compared with control teeth (A). **Pulp spaces reconstructed via** 3D visualization of RO teeth (B, C and D) compared with the control (A). Abbreviations: SEM, scanning electron microscopy; RO, Regional odontodysplasia.

The root structures showed marked hypoplasia, irregular conical shapes, and open apices. The root length was reduced by approximately 40 % for tooth 35, 90 % for tooth 36, and more than 95 % for tooth 37. Pulp spaces were considerably reduced, resulting in a decrease in volume of 50 %–70 % compared with that of the control teeth. These findings are consistent with the hypoplastic nature of enamel and dentin formation in ROs (Fig. 2).

### 4.2 Scanning electron microscopy analysis

Scanning electron microscopy (SEM) provides high-resolution visualization of enamel and dentin structures. Tooth 36 presented surface depressions or “pits” indicative of mineralization defects, whereas lacunae were prominent on tooth 35, suggesting areas of resorption. Magnifications ranging from × 10 to × 1000 revealed well-defined enamel prisms and internal dentin structures in both control and affected teeth, indicating that the enamel phase remained mineralized despite morphological anomalies.

**Fig. 3:**
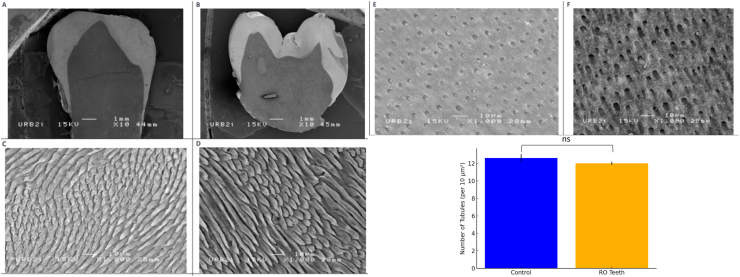
**Comparative SEM images of control and RO teeth:** Scanning electron microscopy (SEM) images of control (A, C, E) and affected (B, D, F) teeth following acid etching. (A) and (B): General view of enamel and dentin at × 10 magnification. (C) and (D): High-magnification view of enamel at × 1000 magnification. (E) and (F): High-magnification view of the coronal dentin at × 1000 magnification. (G): Comparison of the number of coronal dentinal tubes between affected teeth and control teeth.

A comparison of the number of coronal dentinal tubules between affected and healthy teeth revealed a slight nonsignificant decrease in the number of dentinal tubules in teeth affected by RO. Additionally, the dentinal tubules in the affected teeth were relatively wider than those in healthy controls were, further highlighting structural differences at the microscopic level. The dentin displayed widened dentinal tubules with irregular distributions, which was consistent with inte-rtubular dentin patterns. The application of phosphoric acid improved the visualization of enamel and dentin boundaries, confirming the structural presence of hydroxyapatite crystallites. Despite surface irregularities, no significant disruptions were observed in the intrinsic organization of the dentin or enamel (Fig. 3).

### 4.3 Mineral density analysis

**Fig. 4:**
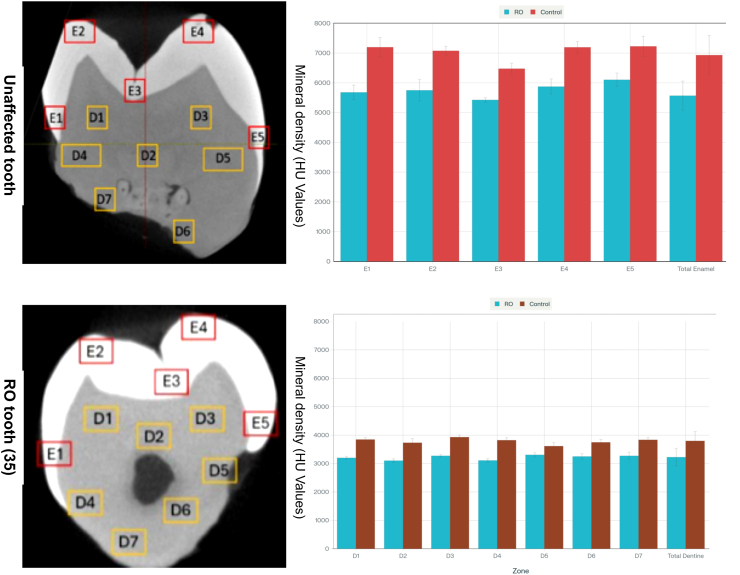
Comparison of mineral density between RO teeth and control teeth measured within the enamel and dentinal regions of interest (ROIs). *Abbreviations: RO, Regional odontodysplasia;* ROIs, regions of interest.

Mineral density was assessed via micro-CT imaging (AnalyzeDirect software, version 14.0). The objective of this analysis was not to quantify absolute hydroxyapatite concentration but rather to explore mineralization defects in the affected teeth. Densities for each tooth were obtained in **mean** Hounsfield Units (HU): for dentin, 3797.9 HU (tooth 35), 3499.0 HU (tooth 36), and 3791.6 HU (tooth 37), compared with a mean value of 3766.8 HU for normal teeth. For enamel, 6931.0 HU (tooth 35), 6070.3 HU (tooth 36), and 6579.4 HU (tooth 37). Analysis across regions of interest (ROIs) revealed a uniform mineral density distribution, with no significant intra- or inter-tooth variations. In contrast to **many** published case reports of regional odontodysplasia, which highlight prominent hypomineralization, **These affected teeth analysed with** micro-CT analysis **present** no significant reduction of enamel or dentin mineral density in the affected teeth when compared to literature-reported values for healthy tissue evaluated with the same methodology ^8^**.** The observed homogeneous mineralization pattern, despite pronounced morphological defects argues against a generalized **under** developmental or inflammatory hypomineralization. These findings are visually summarized in Fig. 4 (HU values) and **raw data are available** in Supplementary Material 2.

## 5 Discussion

The clinical and radiographic characteristics of this case align partially with established findings in the literature. Eruption disturbances, crown and root anomalies, but not mineralization and the quality of dental tissues (enamel and dentin). RO typically manifests as a localized developmental anomaly predominantly affecting the maxilla (70 %) and often presents unilaterally.^8^^,^^9^ In this case, however, the mandibular left quadrant was involved, an atypical presentation supported by isolated reports.^10^^,^^11^

Previous reports of mandibular RO, such as those by Al-Tuwirqi et al., ^7^ Ji et al., ^6^ and Bakerywala et al. ^10^^,^^11^, also described unilateral involvement with varying degrees of root hypoplasia and delayed eruption. The present case aligns with these features but provides an additional microstructural and mineral assessment that has rarely been reported for mandibular presentations. ^6^^,^^7^^,^^10^

Although regional odontodysplasia has been described for several decades, many aspects of its pathogenesis remain unclear. In particular, it is still unknown whether RO represents a strictly developmental anomaly or whether inflammatory resorption processes contribute to its onset. Furthermore, few studies have quantitatively assessed the mineral density of affected enamel and dentin, leaving unanswered whether the fragility of RO teeth results from hypomineralization or from microstructural disorganization. Moreover, it remains unclear why the alveolar bone is preserved despite severe root dysplasia, perhaps suggesting a localized rather than systemic lesion. The present study was therefore designed to address these gaps by combining micro-computed tomography to assess three-dimensional morphology and mineral density, scanning electron microscopy to characterize microstructural organization, and quantitative mineral analysis to evaluate compositional integrity. This integrative approach was intended to clarify the structural and compositional basis of RO and to identify possible links between developmental and inflammatory processes.

A significant reduction in root length was observed: 40 % for tooth 35, 90 % for tooth 36, and more than 95 % for tooth 37. These anomalies are consistent with findings from other RO studies that reported arrested or altered root development.^11^ Despite severe root dysplasia, the alveolar bone height appeared preserved. This finding raises the possibility that resorption processes, potentially influenced by local inflammatory changes, could contribute to the observed root alterations.^12^ Moreover, agenesis of tooth 38 was consistent with the literature, which frequently documents developmental disturbances in teeth adjacent to the affected area.^12^ The discrepancies between mineral density and structural findings emphasize the multifactorial nature of RO pathogenesis. The preservation of alveolar bone height despite severe root dysplasia suggests that the roots may undergo inflammatory rhizolysis (the expression of matrix metalloproteins), possibly triggered by localized vasculopathy or subclinical infection.^13^^,^^14^ The presence of wide dentinal tubules and resorption lacunae further supports this hypothesis. However, no clinical or laboratory evidence of systemic or localized inflammatory abnormalities was observed in this patient.

The dentinal tubules in the affected teeth were irregular, less numerous, and wider than those in the unaffected teeth. This finding correlates with findings previously reported.^11^^,^^15^ Widened dentinal tubules facilitate bacterial ingress, predisposing teeth to pulpitis and necrosis. Enlarged dentinal tubules and their increased density align with studies on the microstructure of RO dentin, highlighting these structures as potential markers for inflammatory resorption.^9^^,^^12^

Despite preservation of enamel prisms and intertubular dentin, the irregular architecture and reduced root formation likely contribute to the overall fragility of the affected teeth.^16^

Mineral density analysis revealed no significant differences between the affected teeth and controls at different regions in the enamel and dentin. The coronal enamel density values ranged from 6400 to 6800 mg/mm^3^, and the dentin density values ranged from 3600 to 3800 mg/mm^3^, which are consistent with the normal ranges reported in previous studies.^17^ The preserved mineral density contrasts with the clinical manifestation of morphological changes, suggesting that the structural fragility observed in RO may be more attributable to localized morphological developmental anomalies rather than systemic mineralization deficiencies.

Interestingly, while the histological and SEM findings highlighted hypoplastic enamel and dentin anomalies, the mineral content analysis did not reveal corresponding deficits. These findings suggest that functional impairment may be derived from microstructural organization rather than composition. These findings support previous studies indicating the importance of structural integrity over mineral content in determining dental tissue resilience. ^8^^,^^16^

While some studies suggest a link between hypomineralization and systemic disturbances, the preserved mineral density in this case supports localized developmental or inflammatory etiologies.^8^ The observation of wide, irregular dentinal tubules corroborates findings from Alves et al. ^12^ and Mabrouk et al., ^9^ who associated these features with increased susceptibility to infections and resorption.^9^^,^^12^

The preserved enamel and dentin mineral content despite structural anomalies parallels findings by Ide et al., ^16^ who noted that mineralization stages might not correlate directly with morphological defects. This insight has significant implications for future research, particularly in exploring the molecular pathways governing tissue formation and resorption in Ros.^16^

This study underscores the importance of integrating clinical, morphological, and microstructural analyses to understand the RO fully. Future research should explore the inflammatory markers and genetic pathways involved in RO, particularly in patients with atypical presentations such as this one. The findings also highlight the challenges in managing RO cases. Early diagnosis via advanced imaging modalities such as CBCT is crucial for precise morphological assessment and treatment planning.^9^ Prosthetic rehabilitation must consider preserved alveolar bone, which provides a favourable prognosis for implant placement, as reported in similar cases. ^18^

### 5.1 Preservation of alveolar bone: mechanistic implications

The preserved alveolar bone height despite severe root dysplasia represents a distinctive and clinically favourable feature. This observation suggests that bone resorption processes may be decoupled from root morphogenesis in RO, or that systemic factors governing bone metabolism remain unaffected. This finding has favourable implication for prosthetic rehabilitation and implant-supported restoration, as documented in similar cases ^8^^,^^19^

### 5.2 Synthesis and limitations

This integrated analysis suggests that structural fragility in RO derives from localized developmental anomalies affecting macroscopic crown-root morphology and microscopic tissue organization, rather than from systemic mineralization deficiency. The preservation of alveolar bone, despite severe root dysplasia, implies that RO may represent a localized developmental disturbance, potentially with secondary inflammatory sequelae, rather than a primary mineralization or inflammatory disorder.

Several limitations constrain interpretation of these findings. As a single-case report, generalization to all RO presentations is inappropriate. The absence of molecular, genetic, or histochemical investigation precludes definitive mechanistic inference regarding the contribution of inflammatory pathways or hereditary factors.

### 5.3 Future directions

Advancing understanding of RO pathogenesis will require multi-modal approaches: molecular analysis of inflammatory markers and osteoclastic mediators, genetic sequencing to identify heritable predisposition, and prospective longitudinal assessment in cohorts of affected patients. High-resolution imaging modalities (micro-CT, CBCT with metal artifact reduction) combined with molecular histology may elucidate the interplay between developmental and inflammatory mechanisms. ^14^^,^^20^ From a clinical perspective, the present case emphasizes that early diagnosis via advanced imaging enables precise morphologic characterization and treatment planning, and that preservation of alveolar bone creates favourable conditions for prosthetic rehabilitation and implant-supported restoration.

## Acknowledgements author contributions

OC, CB, AN, and ES drafted and wrote the manuscript.

ES managed literature review of the introduction.

LF, ES, BVF, and AN managed the clinical case.

OC and AN performed the experiments.

OC, CB and AN prepared the figures.

PG, LF, CB and BVF provided critical revision of the manuscript. Writing, Figure preparation.

## Funding

This research received no external funding.

## Declaration of competing interest

The authors declare that they have no known competing financial interests or personal relationships that could have appeared to influence the work reported in this paper.
