# Supplementary material for: Regional odontodysplasia: a report of unusual case with in-depth analysis
Source: J Oral Biol Craniofac Res. 2026 Jan 15;16(2):101394. doi: 10.1016/j.jobcr.2025.12.012 (PMC12830254; doi:10.1016/j.jobcr.2025.12.012)
Supplement: Multimedia component 1 [file mmc1.pdf]

Micro CT Protocol

Application: Analyze 14.0

Create folder

I. Imput/output

1. 1 Open folder Z (see photo 1).
2. Select the first image, then hold Shift (up arrow) and select the last image.
3. Click the mouse wheel and move the mouse to scroll through the images and check that there is no blur.
4. Click on Max/Min (at the bottom right of the screen), then click on an image and move the mouse to adjust the contrast.
5. Click on Load Volume at the bottom left of the screen.
6. Exit (bottom right).

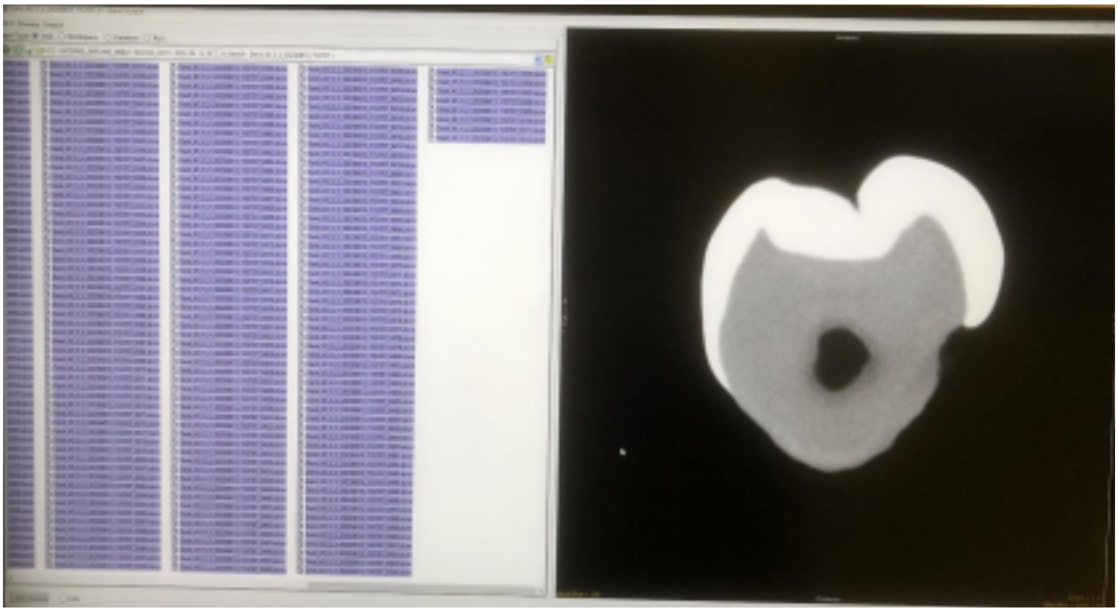

II. Display

1) Rien à faire je crois dans ce paramètre (c'est pour l'orientation/sections)

III. Process

1. Click on "Spatial Filter," then "Median," then set "Kernel Size"—often at 5, 5, 5, but here use 3, 3, 3 so that the grain is identical between the two images.

(If there is a problem with contrast between the two images, adjust min and max to match. Use the mouse upward for max and left or right for min.)

2. Click on "Process Volume."

3. Click on "Save Volume."

4. Exit this screen by quitting at the top right.

5. (You can view information about your image by right click + info)

6. Click on the image and drag it into a Workspace (1), then rename it "filtered" (since the filter has been applied) with right click > Rename Workspace.

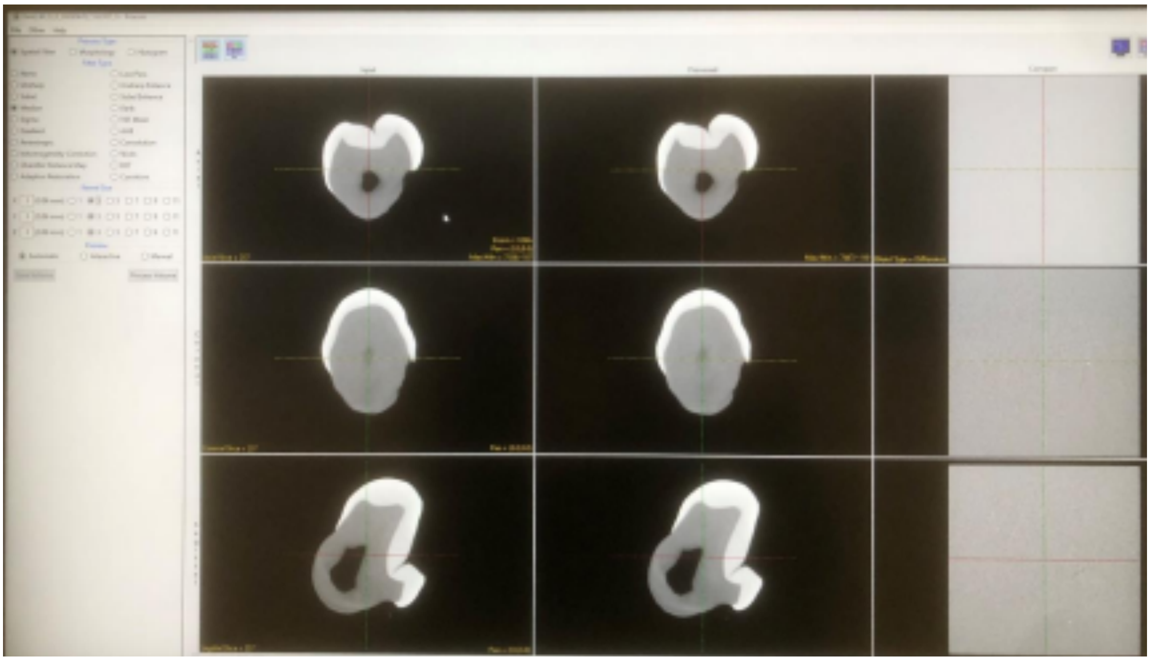

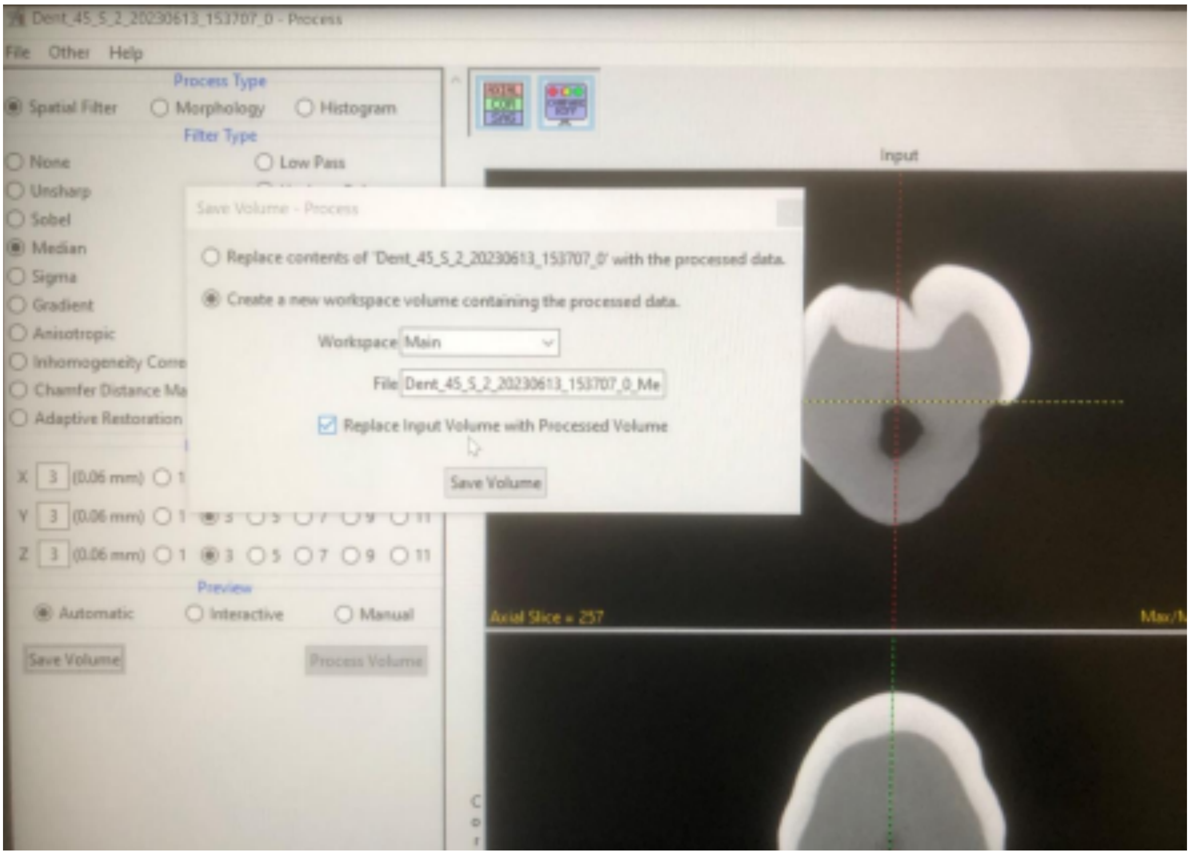

IV. Segmentation

1. Select/click on the image in the menu, then choose “Segment.” You will arrive at the “Edge Strength” step.

2. Choose “semi-automatic.”

3. Select “Threshold Volume.”

4. Adjust the Threshold bar, setting only the minimum value (here, 4579). Note that all sample thresholds must be identical for each image.

5. Threshold object: enamel.

6. Set edge strength to 586 on the scale.

7. Lock enamel (click the padlock icon at the lower left of “enamel”).

8. Repeat for dentin: add object -> threshold volume -> 2782 -> threshold object -> lock enamel.

9. Click “Use Edge Strength to Enhance segmentation.”

10. Adjust the “Edge” scale to separate tissues (here, 586). Always use the same setting (667).

11. Region Grow (next to Threshold Volume).

12. Click on the lower tooth part “enamel” for example. A cross appears on both the lower and upper tooth.

13. Click “Connect Object” and wait

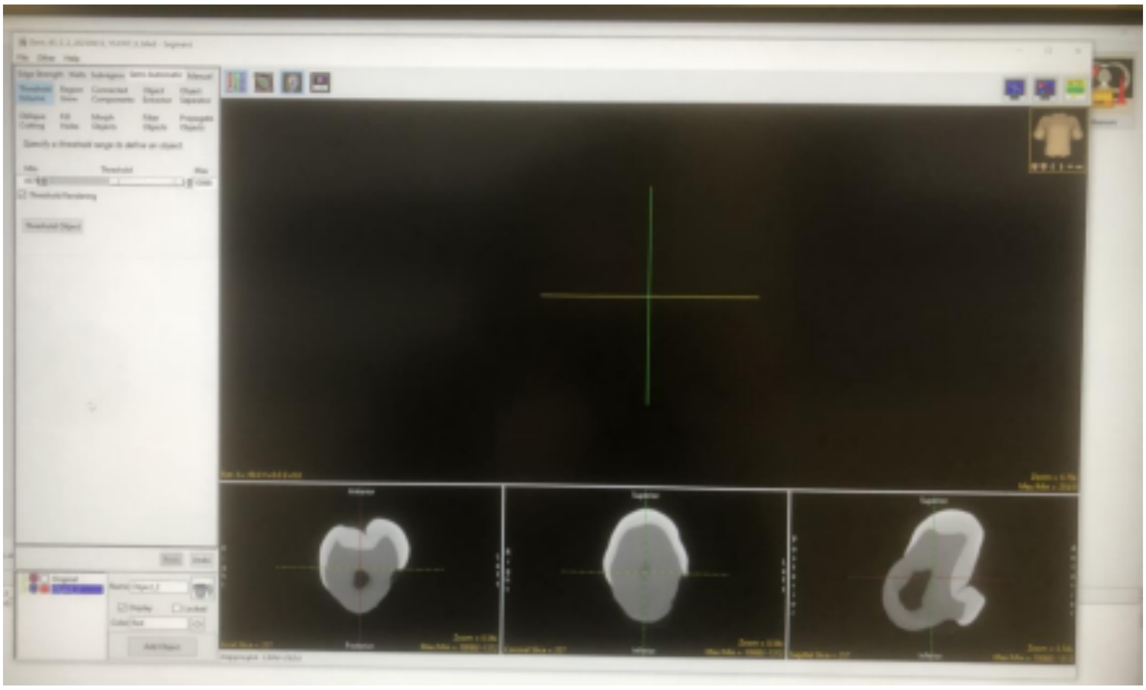

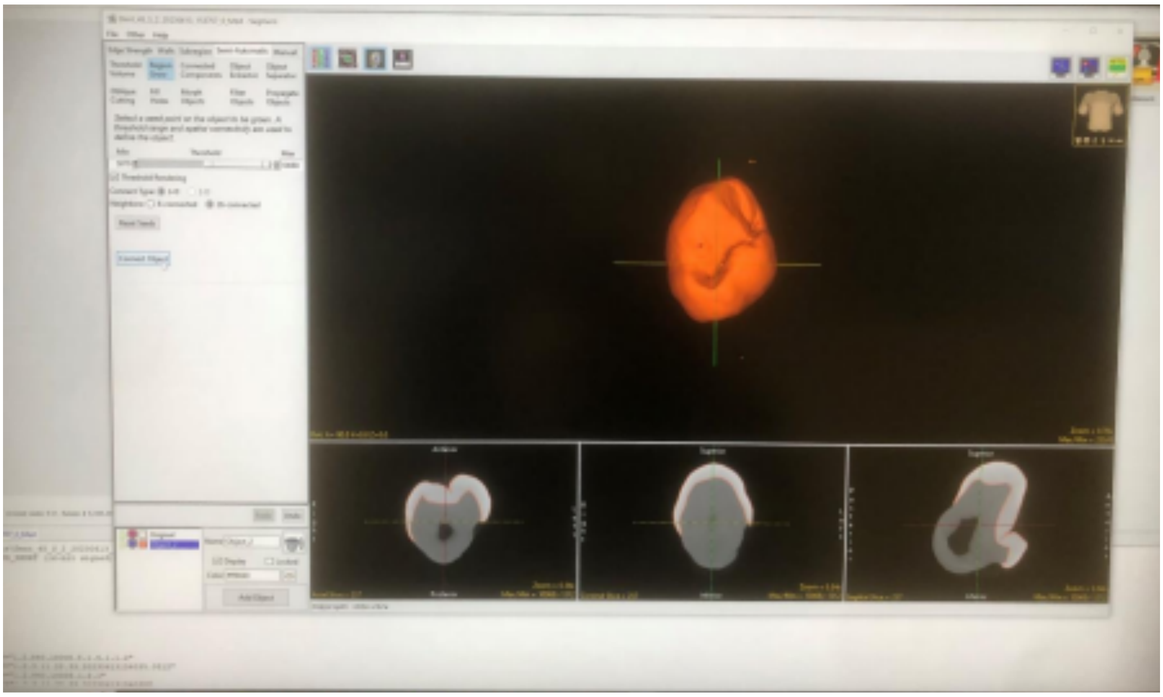

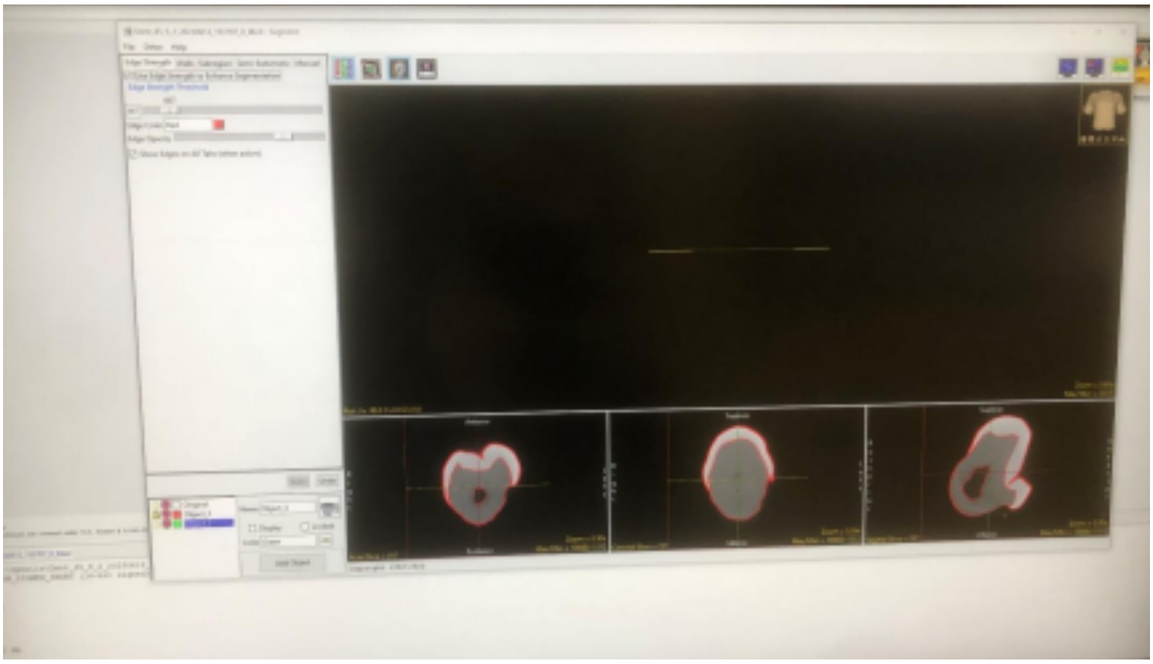

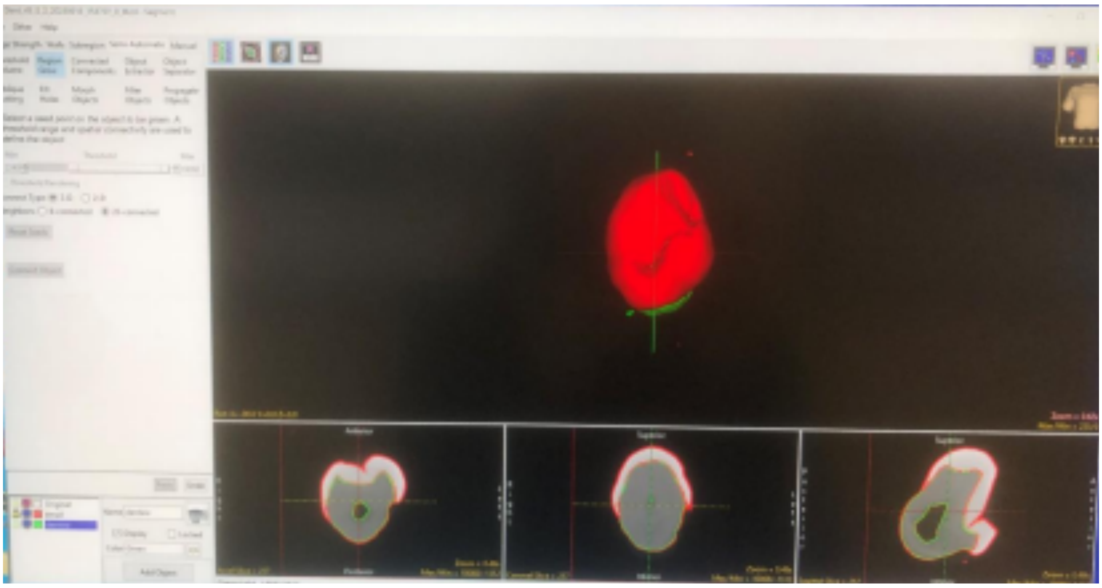

V. Measure

1) Select Image
2) Measure
3) 3D
4) Sample Enabled Objects
5) Size Intensity
6) Cocher « St. Dev. »
7) Log Stats

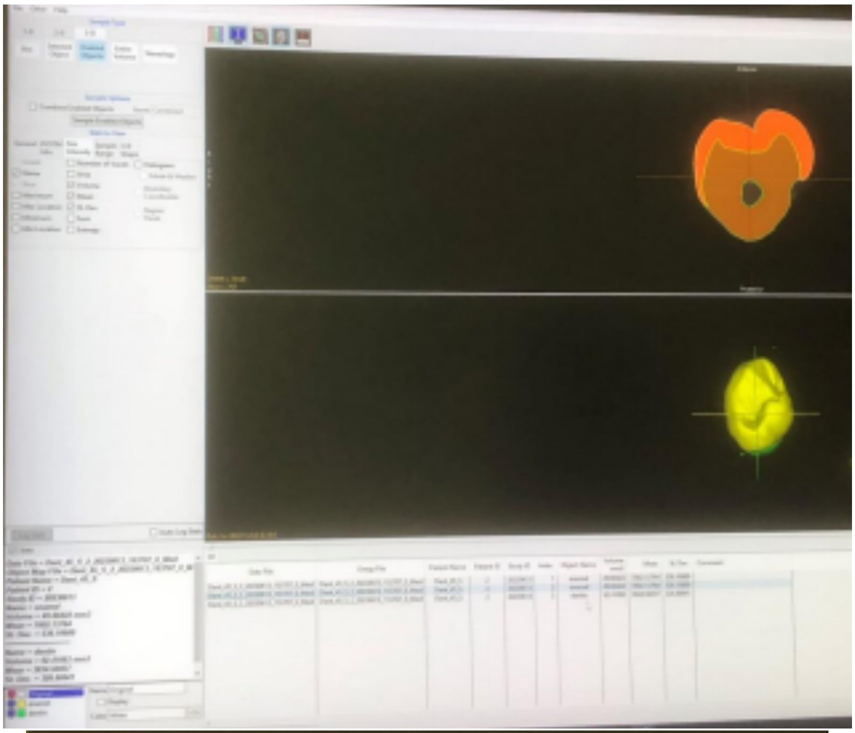

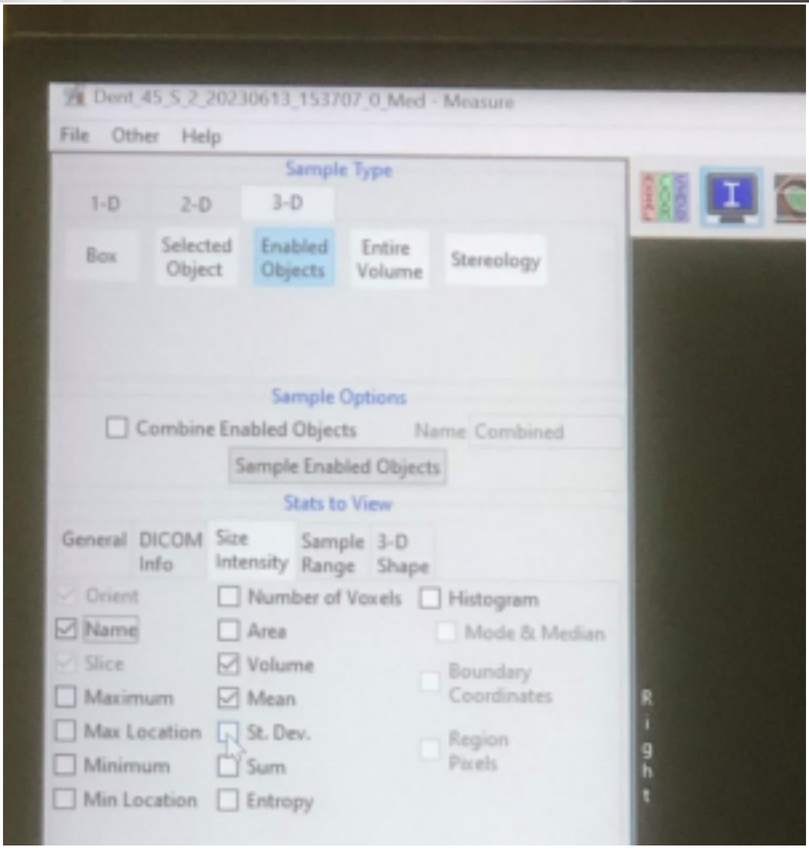

Mineral Density Analysis

1. Copy and paste the statistics table obtained from Analyze 14.0 software, including Volume / Mean / Standard Deviation into Excel.
2. Retrieve the phantom calibration Excel file and take the equation from the graph on the right. Replace the variable x by the obtained mean values.
3. Replace decimal points by commas.

Image densities were scaled in Hounsfield Units (HU) during acquisition and converted to mineral density (mgHA cm−3)
