# Supplementary material for: Regional odontodysplasia: a report of unusual case with in-depth analysis
Source: J Oral Biol Craniofac Res. 2026 Jan 15;16(2):101394. doi: 10.1016/j.jobcr.2025.12.012 (PMC12830254; doi:10.1016/j.jobcr.2025.12.012)
Supplement: Multimedia component 2 [file mmc2.pdf]

Tooth 35

Data File	Omap File	Stu Index		Object Name
Dent_35_S_2_filtered	Dent_35_S_2_filtered	##	1	émail
Dent_35_S_2_filtered	Dent_35_S_2_filtered	##	2	dentine
Dent_35_S_2_filtered	Dent_35_S_2_filtered	##	3	D1
Dent_35_S_2_filtered	Dent_35_S_2_filtered	##	4	D3
Dent_35_S_2_filtered	Dent_35_S_2_filtered	##	5	D2
Dent_35_S_2_filtered	Dent_35_S_2_filtered	##	6	D4
Dent_35_S_2_filtered	Dent_35_S_2_filtered	##	7	D6
Dent_35_S_2_filtered	Dent_35_S_2_filtered	##	8	D5
Dent_35_S_2_filtered	Dent_35_S_2_filtered	##	9	D7
Dent_35_S_2_filtered	Dent_35_S_2_filtered	##	10	E2
Dent_35_S_2_filtered	Dent_35_S_2_filtered	##	11	E3
Dent_35_S_2_filtered	Dent_35_S_2_filtered	##	12	E4
Dent_35_S_2_filtered	Dent_35_S_2_filtered	##	13	E5
Dent_35_S_2_filtered	Dent_35_S_2_filtered	##	14	E1

Tooth 36

Data File	Omap File	Stu Index		Object Name
Dent_36_1_filtered0	Dent_36_1_filtered0	##	1	émail
Dent_36_1_filtered0	Dent_36_1_filtered0	##	2	dentine
Dent_36_1_filtered0	Dent_36_1_filtered0	##	3	D1
Dent_36_1_filtered0	Dent_36_1_filtered0	##	4	D3
Dent_36_1_filtered0	Dent_36_1_filtered0	##	5	D2
Dent_36_1_filtered0	Dent_36_1_filtered0	##	6	D4
Dent_36_1_filtered0	Dent_36_1_filtered0	##	7	D5
Dent_36_1_filtered0	Dent_36_1_filtered0	##	8	D6
Dent_36_1_filtered0	Dent_36_1_filtered0	##	9	D7
Dent_36_1_filtered0	Dent_36_1_filtered0	##	10	E1
Dent_36_1_filtered0	Dent_36_1_filtered0	##	11	E2
Dent_36_1_filtered0	Dent_36_1_filtered0	##	12	E4
Dent_36_1_filtered0	Dent_36_1_filtered0	##	13	E3
Dent_36_1_filtered0	Dent_36_1_filtered0	##	14	E5

Tooth 36

Data File	Omap File	Stu Index		Object Name
Dent_36_2_filtered0	Dent_36_2_filtered0	##	1	émail
Dent_36_2_filtered0	Dent_36_2_filtered0	##	2	dentine
Dent_36_2_filtered0	Dent_36_2_filtered0	##	3	D1
Dent_36_2_filtered0	Dent_36_2_filtered0	##	4	D3
Dent_36_2_filtered0	Dent_36_2_filtered0	##	5	D2
Dent_36_2_filtered0	Dent_36_2_filtered0	##	6	D4
Dent_36_2_filtered0	Dent_36_2_filtered0	##	7	D5
Dent_36_2_filtered0	Dent_36_2_filtered0	##	8	D7
Dent_36_2_filtered0	Dent_36_2_filtered0	##	9	D6
Dent_36_2_filtered0	Dent_36_2_filtered0	##	10	E3
Dent_36_2_filtered0	Dent_36_2_filtered0	##	11	E2
Dent_36_2_filtered0	Dent_36_2_filtered0	##	12	E4
Dent_36_2_filtered0	Dent_36_2_filtered0	##	13	E1
Dent_36_2_filtered0	Dent_36_2_filtered0	##	14	E5

Tooth 37

Data File	Omap File	Stu Index		Object Name
Dent_37_3_filtered	Dent_37_3_filtered	##	1	émail
Dent_37_3_filtered	Dent_37_3_filtered	##	2	dentine
Dent_37_3_filtered	Dent_37_3_filtered	##	3	D1

Dent_37_3_filtered	Dent_37_3_filtered	##	4	D3
Dent_37_3_filtered	Dent_37_3_filtered	##	5	D2
Dent_37_3_filtered	Dent_37_3_filtered	##	6	D4
Dent_37_3_filtered	Dent_37_3_filtered	##	7	D5
Dent_37_3_filtered	Dent_37_3_filtered	##	8	D6
Dent_37_3_filtered	Dent_37_3_filtered	##	9	D7
Dent_37_3_filtered	Dent_37_3_filtered	##	10	E1
Dent_37_3_filtered	Dent_37_3_filtered	##	11	E5
Dent_37_3_filtered	Dent_37_3_filtered	##	12	E3
Dent_37_3_filtered	Dent_37_3_filtered	##	13	E2
Dent_37_3_filtered	Dent_37_3_filtered	##	14	E4

Normal value control of 36

Data File	Omap File	Stu Index		Object Name
A_6_20230710_151254_0_M	A_6_20230710_151254_0_Med	##	1	émail
A_6_20230710_151254_0_M	A_6_20230710_151254_0_Med	##	2	dentine
A_6_20230710_151254_0_M	A_6_20230710_151254_0_Med	##	3	E1
A_6_20230710_151254_0_M	A_6_20230710_151254_0_Med	##	4	E5
A_6_20230710_151254_0_M	A_6_20230710_151254_0_Med	##	5	E3
A_6_20230710_151254_0_M	A_6_20230710_151254_0_Med	##	6	E2
A_6_20230710_151254_0_M	A_6_20230710_151254_0_Med	##	7	E4
A_6_20230710_151254_0_M	A_6_20230710_151254_0_Med	##	8	D1
A_6_20230710_151254_0_M	A_6_20230710_151254_0_Med	##	9	D3
A_6_20230710_151254_0_M	A_6_20230710_151254_0_Med	##	10	D2
A_6_20230710_151254_0_M	A_6_20230710_151254_0_Med	##	11	D4
A_6_20230710_151254_0_M	A_6_20230710_151254_0_Med	##	12	D5
A_6_20230710_151254_0_M	A_6_20230710_151254_0_Med	##	13	D6
A_6_20230710_151254_0_M	A_6_20230710_151254_0_Med	##	14	D7

Normal sample

Data File	Omap File	Stu Index		Object Name
P3_51_50_20240115_14400	témoin dentine 51	##	1	émail
P3_51_50_20240115_14400	témoin dentine 51	##	2	dentine
P3_51_50_20240115_14400	témoin dentine 51	##	3	D1
P3_51_50_20240115_14400	témoin dentine 51	##	4	D3
P3_51_50_20240115_14400	témoin dentine 51	##	5	D2
P3_51_50_20240115_14400	témoin dentine 51	##	6	D4
P3_51_50_20240115_14400	témoin dentine 51	##	7	D5
P3_51_50_20240115_14400	témoin dentine 51	##	8	D6
P3_51_50_20240115_14400	témoin dentine 51	##	9	D7

Volume mm3	Mean	St, Dev,
51,26947	6931,03565	657,37864
61,06118	3797,90947	324,37231
0,06648	3846,30084	64,85037
0,06648	3931,06185	70,61725
0,06648	3734,1207	142,64846
0,06648	3822,21709	86,82137
0,06648	3748,49471	102,16386
0,13296	3614,46089	118,97138
0,06648	3835,67341	80,07956
0,06648	7075,49952	152,22498
0,06648	6475,5	184,22866
0,06648	7193,1432	195,29343
0,06648	7226,19735	338,5316
0,02904	7196,50496	325,00381

Volume mm3	Mean	St, Dev,
167,97718	6070,31472	612,19532
324,62394	3498,99359	227,33642
0,06648	3459,94332	88,02423
0,06648	3450,95644	85,38832
0,06648	3193,69844	100,14782
0,06648	3548,32214	72,79197
0,06648	3453,18339	84,91066
0,06648	3503,20301	79,46391
0,06648	3604,54091	177,69774
0,06648	6639,96258	447,49996
0,06648	6158,00566	142,73704
0,13296	5719,3299	326,25321
0,06648	5367,08833	109,51964
0,06648	6660,00662	326,31354

Volume mm3	Mean	St, Dev,
196,67891	5725,99088	532,97314
343,1319	3177,41726	256,0877
0,53184	3177,67148	71,56673
0,53184	3064,92864	60,53547
0,53184	2928,91252	121,02125
0,53184	3268,82684	129,21719
0,53184	3269,57942	86,19202
0,53184	3149,54188	71,11186
0,53184	3223,66414	60,62097
0,53184	5172,93574	93,86204
0,53184	5706,60698	221,77153
0,53184	5656,8219	146,57047
0,53184	6028,00505	370,20096
0,53184	6028,74465	301,41805

Volume mm3	Mean	St, Dev,
73,2721	6579,43535	644,35695
67,32508	3791,64529	318,60458
0,06648	3706,86787	115,84224

0,06648	3815,42623	113,02793
0,06648	3814,67401	85,86977
0,06648	3846,19398	105,16848
0,06648	3692,158	94,12279
0,06648	3754,06101	278,84348
0,06648	3876,24729	129,77061
0,06648	6930,29591	365,01199
0,06648	6993,98099	242,79653
0,06648	6298,92238	123,84263
0,06648	6808,14428	144,47825
0,06648	6727,2444	226,37909

Volume mm3	Mean	St, Dev,
71,68179	5569,95411	485,71031
191,95526	3223,68081	303,02185
0,53184	5680,40999	241,68318
0,53184	6103,76751	223,21829
0,53184	5427,7	77,97585
0,53184	5749,90698	363,10827
0,53184	5874,35945	255,63647
0,53184	3200,20012	53,13325
0,53184	3272,12022	48,48964
0,53184	3101,05644	63,82007
0,53184	3107,5781	62,99325
0,53184	3308,82948	77,48619
0,53184	3248,92142	98,54238
0,53184	3271,33454	129,18908

Volume mm3	Mean	St. Dev.
6.60594	6553.16698	855.94429
60.01492	3766.75602	389.73272
0.06648	3647.83478	90.17388
0.06648	3786.05921	100.81103
0.06648	3522.86510	155.78390
0.06648	3669.00734	83.99901
0.06648	3711.70481	75.52064
0.06648	3747.35102	110.70477
0.06648	3893.82816	89.80928

Data File	Omap File	I I	Index	Object Name
Dent_35_S_2_filtered	Dent_35_S_2_filtered2	I	1	émail
Dent_35_S_2_filtered	Dent_35_S_2_filtered2	I	2	dentine
Dent_35_S_2_filtered	Dent_35_S_2_filtered2	I	3	D1
Dent_35_S_2_filtered	Dent_35_S_2_filtered2	I	4	D3
Dent_35_S_2_filtered	Dent_35_S_2_filtered2	I	5	D2
Dent_35_S_2_filtered	Dent_35_S_2_filtered2	I	6	D4
Dent_35_S_2_filtered	Dent_35_S_2_filtered2	I	7	D5
Dent_35_S_2_filtered	Dent_35_S_2_filtered2	I	8	D6
Dent_35_S_2_filtered	Dent_35_S_2_filtered2	I	9	D7
Dent_35_S_2_filtered	Dent_35_S_2_filtered2	I	10	E1
Dent_35_S_2_filtered	Dent_35_S_2_filtered2	I	11	E5
Dent_35_S_2_filtered	Dent_35_S_2_filtered2	I	12	E2
Dent_35_S_2_filtered	Dent_35_S_2_filtered2	I	13	E4
Dent_35_S_2_filtered	Dent_35_S_2_filtered2	I	14	E3

Data File	Omap File	I I	Index	Object Name
Dent_36_1_filtered0	Dent_36_1_filtered02	I	1	émail
Dent_36_1_filtered0	Dent_36_1_filtered02	I	2	dentine
Dent_36_1_filtered0	Dent_36_1_filtered02	I	3	D1
Dent_36_1_filtered0	Dent_36_1_filtered02	I	4	D3
Dent_36_1_filtered0	Dent_36_1_filtered02	I	5	D2
Dent_36_1_filtered0	Dent_36_1_filtered02	I	6	D4
Dent_36_1_filtered0	Dent_36_1_filtered02	I	7	D5
Dent_36_1_filtered0	Dent_36_1_filtered02	I	8	D6
Dent_36_1_filtered0	Dent_36_1_filtered02	I	9	D7
Dent_36_1_filtered0	Dent_36_1_filtered02	I	10	E1
Dent_36_1_filtered0	Dent_36_1_filtered02	I	11	E5
Dent_36_1_filtered0	Dent_36_1_filtered02	I	12	E2
Dent_36_1_filtered0	Dent_36_1_filtered02	I	13	E4
Dent_36_1_filtered0	Dent_36_1_filtered02	I	14	E3

Data File	Omap File	I I	Index	Object Name
Dent_36_2_filtered0	Dent_46_2_filtered2	I	1	émail
Dent_36_2_filtered0	Dent_46_2_filtered2	I	2	dentine
Dent_36_2_filtered0	Dent_46_2_filtered2	I	3	D1
Dent_36_2_filtered0	Dent_46_2_filtered2	I	4	D3
Dent_36_2_filtered0	Dent_46_2_filtered2	I	5	D2
Dent_36_2_filtered0	Dent_46_2_filtered2	I	6	D4
Dent_36_2_filtered0	Dent_46_2_filtered2	I	7	D5
Dent_36_2_filtered0	Dent_46_2_filtered2	I	8	D6
Dent_36_2_filtered0	Dent_46_2_filtered2	I	9	D7
Dent_36_2_filtered0	Dent_46_2_filtered2	I	10	E1
Dent_36_2_filtered0	Dent_46_2_filtered2	I	11	E5
Dent_36_2_filtered0	Dent_46_2_filtered2	I	12	E2
Dent_36_2_filtered0	Dent_46_2_filtered2	I	13	E4
Dent_36_2_filtered0	Dent_46_2_filtered2	I	14	E3

Data File	Omap File	I I	Index	Object Name
Dent_37_3_filtered	Dent_37_3_filtered2	I	1	émail
Dent_37_3_filtered	Dent_37_3_filtered2	I	2	Dentine
Dent_37_3_filtered	Dent_37_3_filtered2	I	3	D1

Dent_37_3_filtered	Dent_37_3_filtered2	I	4	D3
Dent_37_3_filtered	Dent_37_3_filtered2	I	5	D2
Dent_37_3_filtered	Dent_37_3_filtered2	I	6	D4
Dent_37_3_filtered	Dent_37_3_filtered2	I	7	D5
Dent_37_3_filtered	Dent_37_3_filtered2	I	8	D6
Dent_37_3_filtered	Dent_37_3_filtered2	I	9	D7
Dent_37_3_filtered	Dent_37_3_filtered2	I	10	E1
Dent_37_3_filtered	Dent_37_3_filtered2	I	11	E5
Dent_37_3_filtered	Dent_37_3_filtered2	I	12	E2
Dent_37_3_filtered	Dent_37_3_filtered2	I	13	E4
Dent_37_3_filtered	Dent_37_3_filtered2	I	14	E3

Normal values

Data File	Omap File	I I	Index	Object Name
Sample 32 Temoin 63_16_2023	Sample 32 Temoin 63_16_20231215 S		1	émail
Sample 32 Temoin 63_16_2023	Sample 32 Temoin 63_16_20231215 S		2	dentine
Sample 32 Temoin 63_16_2023	Sample 32 Temoin 63_16_20231215 S		3	D1
Sample 32 Temoin 63_16_2023	Sample 32 Temoin 63_16_20231215 S		4	D3
Sample 32 Temoin 63_16_2023	Sample 32 Temoin 63_16_20231215 S		5	D2
Sample 32 Temoin 63_16_2023	Sample 32 Temoin 63_16_20231215 S		6	D4
Sample 32 Temoin 63_16_2023	Sample 32 Temoin 63_16_20231215 S		7	D5
Sample 32 Temoin 63_16_2023	Sample 32 Temoin 63_16_20231215 S		8	D6
Sample 32 Temoin 63_16_2023	Sample 32 Temoin 63_16_20231215 S		9	D7
Sample 32 Temoin 63_16_2023	Sample 32 Temoin 63_16_20231215 S		10	E1
Sample 32 Temoin 63_16_2023	Sample 32 Temoin 63_16_20231215 S		11	E5
Sample 32 Temoin 63_16_2023	Sample 32 Temoin 63_16_20231215 S		12	E3
Sample 32 Temoin 63_16_2023	Sample 32 Temoin 63_16_20231215 S		13	E4
Sample 32 Temoin 63_16_2023	Sample 32 Temoin 63_16_20231215 S		14	E2

normal vlaues for 37

Data File	Omap File	I I	Index	Object Name
témoin 82 émail0	témoin 82 émail0	I	1	émail
témoin 82 émail0	témoin 82 émail0	I	2	dentine
témoin 82 émail0	témoin 82 émail0	I	3	E1
témoin 82 émail0	témoin 82 émail0	I	4	E5
témoin 82 émail0	témoin 82 émail0	I	5	E2
témoin 82 émail0	témoin 82 émail0	I	6	E4
témoin 82 émail0	témoin 82 émail0	I	7	E3
témoin 82 émail0	témoin 82 émail0	I	8	D1
témoin 82 émail0	témoin 82 émail0	I	9	D3
témoin 82 émail0	témoin 82 émail0	I	10	D2
témoin 82 émail0	témoin 82 émail0	I	11	D4
témoin 82 émail0	témoin 82 émail0	I	12	D5
témoin 82 émail0	témoin 82 émail0	I	13	D6
témoin 82 émail0	témoin 82 émail0	I	14	D7

Normal Sample

Data File	Omap File	I I	Index	Object Name
Sample 32 Temoin 63_16_2023	Sample 32 Temoin 63_16_20231215 S		1	émail
Sample 32 Temoin 63_16_2023	Sample 32 Temoin 63_16_20231215 S		2	dentine

Sample 32 Temoin 63_16_2023	Sample 32 Temoin 63_16_20231215 S	3	D1
Sample 32 Temoin 63_16_2023	Sample 32 Temoin 63_16_20231215 S	4	D3
Sample 32 Temoin 63_16_2023	Sample 32 Temoin 63_16_20231215 S	5	D2
Sample 32 Temoin 63_16_2023	Sample 32 Temoin 63_16_20231215 S	6	D4
Sample 32 Temoin 63_16_2023	Sample 32 Temoin 63_16_20231215 S	7	D5
Sample 32 Temoin 63_16_2023	Sample 32 Temoin 63_16_20231215 S	8	D6
Sample 32 Temoin 63_16_2023	Sample 32 Temoin 63_16_20231215 S	9	D7
Sample 32 Temoin 63_16_2023	Sample 32 Temoin 63_16_20231215 S	10	E1
Sample 32 Temoin 63_16_2023	Sample 32 Temoin 63_16_20231215 S	11	E5
Sample 32 Temoin 63_16_2023	Sample 32 Temoin 63_16_20231215 S	12	E3
Sample 32 Temoin 63_16_2023	Sample 32 Temoin 63_16_20231215 S	13	E4
Sample 32 Temoin 63_16_2023	Sample 32 Temoin 63_16_20231215 S	14	E2

Volume mm3	Mean	St, Dev,
51,23203	6930,5604	657,48374
61,20772	3797,355	323,94316
0,06648	3848,2684	62,88846
0,06648	3903,3105	68,11133
0,06648	3718,2188	151,72148
0,06648	4016,2099	123,04489
0,06648	3574,6079	103,19107
0,06648	3817,2407	109,64546
0,06648	3940,6028	110,86168
0,06648	7209,1887	373,46691
0,06648	7237,5699	294,09395
0,06648	7168,9619	158,81435
0,06648	7267,9853	148,98951
0,06648	6499,8548	83,05042

Volume mm3	Mean	St, Dev,
168,04292	6070,1206	612,28483
324,62387	3498,827	227,28637
0,06648	3398,8303	86,5093
0,06648	3442,1019	80,33963
0,06648	3415,9272	116,45109
0,06648	3730,7265	105,03927
0,06648	3816,9886	171,42742
0,06648	3543,4161	86,95876
0,06648	3680,9801	105,33818
0,06648	6478,1126	256,34573
0,06648	6654,4907	306,72676
0,06648	6145,4149	123,03835
0,06648	6088,594	178,369
0,06648	5342,3708	123,45318

Volume mm3	Mean	St, Dev,
196,67878	5726,1534	532,9176
343,1319	3177,632	256,05574
0,53184	3110,8595	51,45857
0,53184	3051,3095	69,2634
0,53184	2920,7194	126,10448
0,53184	3248,8942	107,58529
0,53184	3262,9885	80,30311
0,53184	3252,7395	71,60286
0,53184	3097,0977	60,94386
0,53184	5881,5301	378,92383
0,53184	6063,3324	297,55307
0,53184	5734,1769	136,52554
0,53184	5763,9013	152,80244
0,53184	5090,4166	71,74231

Volume mm3	Mean	St, Dev,
73,2704	6579,4423	644,32976
67,2605	3796,1321	275,4382
0,06648	3927,8137	100,83535

0,06648	3833,3012	83,34435
0,06648	3914,5061	85,53465
0,06648	3912,0407	165,64172
0,06648	3785,453	144,76895
0,06648	3898,2549	116,01192
0,06648	3822,7835	157,65589
0,06648	7009,5924	288,3688
0,06648	6886,9023	208,18031
0,06648	6793,6093	125,51208
0,06648	6818,0124	231,26287
0,06648	6291,9208	147,08417

Volume mm3	Mean	St, Dev,
29,90322	6707,4153	798,28176
118,30113	3711,2129	357,5104
0,06648	3594,3499	78,42606
0,06648	3654,4099	65,62219
0,06648	3368,0338	93,07023
0,06648	3998,2808	115,97205
0,06648	3852,3681	103,80321
0,06648	3981,6781	77,86201
0,06648	4035,3144	85,02923
0,06648	7073,6274	548,80902
0,06648	7308,3508	269,22047
0,06648	6583,4941	155,65452
0,06648	6538,6726	173,39799
0,06648	7285,2447	206,13475

Volume mm3	Mean	St, Dev,	Comment
133,65096	6377,6254	633,68827	
127,6911	3785,1234	310,59923	
0,06648	6546,2596	329,34909	
0,06648	7055,2635	291,10247	
0,06648	8044,782	219,32872	
0,06648	6696,0205	190,20061	
0,06648	5849,5532	94,1629	
0,06648	3818,348	80,6191	
0,06648	3880,5958	74,83596	
0,06648	3661,2653	99,77207	
0,06648	3901,7496	68,97306	
0,06648	4109,0632	73,08625	
0,06648	4301,2858	150,39488	
0,06648	3672,4489	174,37776	

Volume mm3	Mean	St, Dev,
29,90322	6707,4153	798,28176
118,30113	3711,2129	357,5104

0,06648	3594,3499	78,42606
0,06648	3654,4099	65,62219
0,06648	3368,0338	93,07023
0,06648	3998,2808	115,97205
0,06648	3852,3681	103,80321
0,06648	3981,6781	77,86201
0,06648	4035,3144	85,02923
0,06648	7073,6274	548,80902
0,06648	7308,3508	269,22047
0,06648	6583,4941	155,65452
0,06648	6538,6726	173,39799
0,06648	7285,2447	206,13475
